# Health Information Scanning and Seeking in Diverse Language, Cultural and Technological Media Among Latinx Adolescents: Cross-Sectional Study

**DOI:** 10.2196/64672

**Published:** 2025-03-05

**Authors:** Melissa J DuPont-Reyes, Alice P Villatoro, Lu Tang

**Affiliations:** 1 Departments of Sociomedical Sciences and Epidemiology Columbia University Irving Medical Center New York, NY United States; 2 Department of Public Health Santa Clara University Santa Clara, CA United States; 3 Department of Communication & Journalism Texas A&M University College Station, TX United States

**Keywords:** adolescent behaviors, mental health, Latino, social media, adolescent, media use, internet use, health information seeking, health information scanning, mobile phone

## Abstract

**Background:**

Continuous scientific and policy debate regarding the potential harm and/or benefit of media and social media on adolescent health has resulted, in part, from a deficiency in robust scientific evidence. Even with a lack of scientific consensus, public attitudes, and sweeping social media prohibitions have swiftly ensued. A focus on the diversity of adolescents around the world and their diverse use of language, culture, and social media is absent from these discussions.

**Objective:**

This study aims to guide communication policy and practice, including those addressing access to social media by adolescent populations. This study assesses physical and mental health information scanning and seeking behaviors across diverse language, cultural, and technological media and social media among Latinx adolescent residents in the United States. This study also explores how Latinx adolescents with mental health concerns use media and social media for support.

**Methods:**

In 2021, a cross-sectional survey was conducted among 701 US-based Latinx adolescents aged 13-20 years to assess their health-related media use. Assessments ascertained the frequency of media use and mental and physical health information scanning and seeking across various media technologies (eg, TV, podcasts, and social media) and language and cultural types (ie, Spanish, Latinx-tailored English, and general English). Linear regression models were used to estimate adjusted predicted means of mental and physical health information scanning and seeking across diverse language and cultural media types, net personal and family factors, in the full sample and by subsamples of mental health symptoms (moderate-high vs none-mild).

**Results:**

Among Latinx adolescents, media and social media use was similar across mental health symptoms. However, Latinx adolescents with moderate-high versus none-mild symptoms more often scanned general English media and social media for mental health information (*P*<.05), although not for physical health information. Also, Latinx adolescents with moderate-high versus none-mild symptoms more often sought mental health information on Latinx-tailored and general English media, and social media (*P*<.05); a similar pattern was found for physical health information seeking. In addition, Latinx adolescents with moderate-high versus none-mild symptoms often sought help from family and friends for mental and physical health problems and health care providers for mental health only (*P*<.05).

**Conclusions:**

While media and social media usage was similar across mental health, Latinx adolescents with moderate-high symptoms more often encountered mental health content in general English media and social media and turned to general English- and Latinx-tailored media and social media more often for their health concerns. Together these study findings suggest more prevalent and available mental health content in general English versus Spanish language and Latinx-tailored media and underscore the importance of providing accessible, quality health information across diverse language, cultural, and technological media and social networks as a viable opportunity to help improve adolescent health.

## Introduction

Current reports depict an adolescent mental health crisis in the United States. About 1 in 5 adolescents experience poor mental health, especially race and ethnic minoritized adolescents [1-6]. In 2021, the US Surgeon General stated that the COVID-19 pandemic further altered adolescents’ experiences at home, school, and the community, which generated devastating effects on their mental health [7]. In 2021, 42% of adolescents had persistent sadness and hopelessness and 22% of adolescents considered suicide, representing significant increases since 2019 [8]. Latinx (46%) adolescents, representing 25% of all adolescents nationally, report higher rates of sadness than Asian (35%), Black (39%), and White (41%) adolescents, with the pandemic especially worsening mental health for Latinx adolescents [9]. Despite these trends, less than half of Latinx adolescents with mental health problems receive care [5].

Given the concern about adolescent mental health, emerging public attitudes and policies have focused on how social media in particular may influence mental health outcomes and knowledge, norms, and behaviors related to mental health help-seeking and services [10-21]. While past research has focused on legacy media (eg, TV, radio, print), recent research has examined new digital media, especially social media, as a behavioral risk factor for poor mental health [22]. While the mechanisms explaining this association remain unclear, studies have shown how mental health content on social media is highly variable, portraying both positive (ie, supportive) and negative (ie, stigmatizing) content [17-21,23-28]. For instance, while #MentalHealth is one of the 3 most popular social media tags about health to increase public awareness, pejorative content such as stigma and hate speech is prevalent too [16-21,29,30]. Therefore, legacy and new digital media can deliver mixed mental health content that can potentially influence mental health knowledge, attitudes, behaviors, and outcomes. Thus, further study about adolescents’ exposure to mental health information across diverse languages, cultures, and technological media is warranted, and an assessment of how this exposure might influence mental health behaviors. This knowledge is especially relevant for adolescents as about half of social media users are 10-29 years old—the same age of onset of most mental health problems [23].

Research on legacy media has shown direct links with positive and negative mental health behaviors, while new studies on social media use and mental health among adolescents have only identified small or null associations, partly due to the predominant use of limited assessments of time spent on social media as a simple construct [11,12,19,22,31-33]. This is a substantial limitation of previous studies as social media is not experienced unidimensionally. Rather, diverse linguistic, cultural, and technological social media exists—that is, media across global languages (Spanish, Mandarin, etc); global cultures (eg, Latinx-tailored, Korean pop culture, influencer cult followings, and social movements such as #BlackLivesMatter); and technologies for delivery of communications (social media apps such as YouTube, Facebook, Instagram, Snapchat, TikTok, Sidechat, Pinterest, Reddit; mobile apps; console-, computer-, or VR-based gaming; music, podcasts, film, and TV streaming services such as Netflix, Hulu, Spotify, Pandora, etc) [22]. Thus, previous studies have not considered more robust measurements or algorithm-driven content that is characteristic of social media.

Careful consideration of measurement is important as adolescent populations are diverse, as is their access to language and cultural media across technology. For instance, Latinx populations in the United States use social media more than other races and ethnic groups, including the top 3 popular apps among all adolescents (ie, Instagram, Snapchat, TikTok) [23]. In addition, Latinx adolescents have access to media, including social media, in Spanish and from Latin America due to close global social networks from dynamic migration experiences. At the same time, Latinx communities are impacted by poor-quality, slow dissemination of health communication as industry-led and government-controlled content moderation has historically overlooked Spanish-language and Latinx communications, particularly social media [13-15,34-41].

Because research on media and social media and health has largely focused on English-language media and excluded Latinx representation relative to their share of the demographic of media consumers, new research is warranted that focuses on diverse language, cultural, and technological media among Latinx adolescents and its relationship to health [11,42-45]. To address this gap, the study aimed to generate new knowledge about the frequency of mental and physical health information scanning and seeking across diverse language, cultural, and technological media and test whether these patterns vary by mental health profiles among Latinx adolescents. Because Latinx adolescents’ social media use has been inadequately studied, we importantly use newly validated, culturally appropriate measures to capture diverse language, cultural, and technological media for health information among Latinx adolescents. Our measurement extends previous, traditional measures of media exposure among Latinx populations that focused on language competency and legacy media to now newly capture widely available Latinx-tailored digital media. We also compare these media-based outlets to help-seeking for health problems to family and friends and health care providers.

## Methods

### Sample

In 2021, 701 eligible US-based Latinx adolescents aged 13-20 years from a proprietary Qualtrics survey research panel self-completed a 20-minute cross-sectional web survey using personal digital devices about behaviors related to Spanish- and English-language media use and mental and physical health information acquisition; complete details about study procedures are reported elsewhere [46]. Eligibility included participants identifying as “Latina/o/x/e.” Recruitment quotas by age, ethnic origin, and gender were used to help sample under-represented Latinx groups in population health research. In 2021, smartphone or tablet ownership among US-based adolescents was nearly universal (more than 95%) [47]. Random checks of the survey reviewed accuracy and consistency in data entry. All study materials were offered in Spanish and English; 96.58% (677/701) of the study sample completed the survey in English.

The achieved study sample of Latinx adolescents (N=701) had a median age of 17 (SD 1.57) years; 78.21% (524/701) were female and 86.51% (603/701) were US-born (Table 1). Among the 13.49% (94/701) that were foreign-born, the average age of arrival to the United States was about 10 years old. Ethnic subgroups included Mexican (403/701, 57.49%), Cuban or Dominican or Other Latinx group (83/701, 11.84%), Puerto Rican (74/701, 10.56%), Central American (86/701, 12.27%), and South American (55/701, 7.85%), and race subgroups included American Indian or Alaskan Native (21/701, 3.01%), Black (41/701, 5.88%), Multiracial (74/701, 10.62%), White (244/701, 35.01%), and Other (317/701, 45.48%). About 41% (283/701) reported household incomes of less than US $20,000, while 46% (317/701) reported between US $20,000 and less than US $75,000; 14% (97/701) reported more than US $75,000. Participants reported high Latinx ethnic attachment (mean 33.19, SD 6.86) and English-language media use (mean 3.32, SD 0.94), followed by Latinx-tailored (mean 2.81, SD 1.03) and Spanish-language (mean 2.64, SD 1.05) media use. The study sample compared to national data is reported elsewhere [46]. Subsamples based on the Patient Health Questionnaire-4 (PHQ-4; see Measures section) included no-mild (n=391) and moderate-severe (n=310) mental health symptoms. Subsamples varied significantly by gender, race, parent or guardian nativity, and Spanish acculturation (*P*<.05), and not by other covariates in Table 1.

**Table 1:** Characteristics of Latinx youth full sample and subsamples of mental health symptoms (N=701), 2021.

Characteristics				Full sample (N=701)		No-mild mental health symptoms subsample (n=391)	Moderate-severe mental health symptoms subsample (n=310)				
Age, mean (SD)				17 (1.57)		16.94 (1.55)			17.08 (1.6)		
**Sex, n (%)^a^**											
	Female			524 (78.21)		267 (71.39)	257 (86.82)				
	Male			146 (21.79)		107 (28.61)	39 (13.18)				
**Ethnicity, n (%)**											
	Central American			86 (12.27)		53 (13.55)	33 (10.65)				
	Cuban, Dominican, or Other			83 (11.84)		42 (10.74)	41 (13.23)				
	Mexican			403 (57.49)		229 (58.57)	174 (56.13)				
	Puerto Rican			74 (10.56)		36 (9.21)	38 (12.26)				
	South American			55 (7.85)		31 (7.93)	24 (7.74)				
**Race, n (%)^a^**											
	Black or African American			41 (5.88)		19 (4.91)	22 (7.1)				
	American Indian or Alaskan Native			21 (3.01)		14 (3.62)	7 (2.26)				
	Multiracial			74 (10.62)		31 (8.01)	43 (13.87)				
	Other			317 (45.48)		197 (50.9)	120 (38.71)				
	White			244 (35.01)		126 (32.56)	118 (38.06)				
**Household income, n (%)**											
	US $0-US $19,999			283 (40.6)		152 (39.28)	131 (42.26)				
	US $20,000-US $34,999			134 (19.23)		72 (18.6)	62 (20)				
	US $35,000-US $74,999			183 (26.26)		103 (26.61)	80 (25.81)				
	More than US $75,000			97 (13.92)		60 (15.5)	37 (11.94)				
**Nativity**											
	Foreign-born, n (%)			94 (13.49)		55 (14.21)			39 (12.58)		
	US-born, n (%)			603 (86.51)		332 (85.79)			271 (87.42)		
	Age at arrival, mean (SD)			9.85 (5.44)		10.04 (5.55)			9.59 (5.35)		
	Years lived in the United States, mean (SD)			7.58 (5.35)		7.35 (5.53)			7.9 (5.14)		
	**Parent or guardian**										
		Parent or guardian 1 born in the United States, n (%)^a^	305 (45.12)		155 (41.55)			150 (49.5)			
		Parent or guardian 2 born in the United States, n (%)	261 (39.55)		134 (36.71)			127 (43.05)			
	Latinx ethnic attachment, mean (SD)			33.19 (6.86)		33.21 (6.77)			33.16 (6.98)		
	Spanish acculturation, mean (SD)^a^			25.75 (7.49)		26.26 (7.41)			25.09 (7.55)		
	English acculturation, mean (SD)			33.07 (3.21)		32.97 (3.38)			33.2 (3)		
	Spanish media use, mean (SD)			2.64 (1.05)		2.66 (1.04)			2.62 (1.02)		
	Latinx media use, mean (SD)			2.81 (1.03)		2.84 (1.06)			2.78 (1)		
	English media use, mean (SD)			3.32 (0.94)		3.3 (0.95)			3.33 (0.93)		

^a^*P*<.05 for significant differences across mental health symptoms.

### Measures

#### Media Use (Covariate)

As previously validated, [46] 7 items assessed diverse language, cultural, and technological media types, including Spanish, Latinx-tailored English, and general English media use across print media, free broadcast TV, subscription TV, Twitter, other social media (eg, Facebook, Instagram, and TikTok), radio or podcasts, and music and music streaming. Participants were asked how often they engage in each language, cultural, and technological media type on an average day on a 5-point Likert scale (1=never to 5=always). A previously reported factor analysis [46] revealed that total scores of all items combined for each language or cultural dimension to create three subscales: Spanish-language (α=.92), Latino-tailored English (α=.90), and general English (α=.86) media use.

#### Media-Based Health Information Scanning (Outcome 1)

In total, 5 items assessed the frequency of mental health and, separately, physical health, information scanning (ie, passive exposure to health information in media) across the 3 language or cultural domains (ie, Spanish, Latinx-tailored English, and general English) for each technological media type: print media, TV, websites, social media, or radio or podcasts [46]. Participants were asked how often they encountered (ie, scanning) mental health and, separately, physical health, information in each language, cultural, and technological media type during an average week (1=not at all to 4=a few times a week). Factor analysis [46] revealed that total scores of all 5 media items for each language or cultural domain combined to create 3 subscales of all media scanning for mental health and, separately, for physical health (α=.92-.94).

Furthermore, given recent policy actions related to social media among adolescents, we also examined the 2 items pertaining to social media for each language or cultural domain. Combining the 2 social media items for each language or cultural domain resulted in 3 subscales of social media scanning for mental health and physical health separately.

#### Media-Based Health Information Seeking (Outcome 2)

A total of 5 items assessed the frequency of mental and physical health information seeking (ie, searching on purpose for health information in media) across the 3 language or cultural domains for each technological media type (print media, TV, websites, social media, and radio or podcasts) [46]. Participants were asked about the frequency that they purposely sought mental health and, separately, physical health, information in each media type for a mental or physical health problem of one’s own, a family member, or a friend (1=none to 4=always). Based on the factor analysis that has been previously reported [46], total scores of all items for each language or cultural domain were combined to create 3 subscales of all media information seeking for mental health and, separately, for physical health (α=.87-.90). Also, 2 other configurations of these items by technological media type emerged in factor analyses: a legacy media subscale comprised of items assessing TV, radio, and print media (α=.93; legacy media seeking) and an internet-based media subscale comprised of items assessing websites and social media (α=.89-.90; internet and social media seeking) [46].

#### Formal Health Care and Informal Networks Health Information Seeking (Outcome 3)

A total of 4 items were assessed seeking information for mental health, and separately, a physical health, problem of their own or a family member or a friend, from either health care professionals (formal) or family and friends (eg, in-person or call or text; informal): “For each of the following sources, how often have you actively looked for information about a (physical or mental) health problem that you, a family member, or a friend had?” Participants responded on a 4-point Likert scale (1=never to 4=always) for formal and informal care to the item about mental health followed by a similar item about physical health.

#### Sociodemographic and Mental Health Factors (Other Covariates)

Age, sex, ethnic origin (ie, Latinx country of ancestry), race, nativity (ie, US- or foreign-born), household income, and migration factors (eg, age at arrival to the United States) were measured (Table 1). A total of 6 items assessed ethnic attachment (α=.90), defined as the extent of exploration, belonging, and commitment to one’s Latinx ethnic identity; and 18 items measured acculturation, which captured language use and proficiency (α=.90-.91). Finally, the PHQ-4 screened for mental health symptoms, and demonstrated excellent reliability (α=.89). In total 4 items are scored on a 4-point Likert scale (0=not at all to 3=nearly every day) and summed to 0-12 full scale range. A total of 2 subsamples were created using established cutoff points: scores 0-5=none-mild and scores 6-12=moderate-severe.

### Statistical Analysis

Linear regression models estimated adjusted predicted means of mental and physical health information scanning and seeking across diverse language, cultural, and technological media types (ie, all media inclusive of legacy and internet-based and social media in the Spanish language, Latinx-tailored English language, and general English language). All models were adjusted for age, sex, nativity, race, ethnic origin, household income, ethnic attachment, acculturation, and Spanish, Latinx-tailored English, and general English media use. Models inclusive of the full sample controlled for mental health symptoms. Multicollinearity was not observed between the covariates; all correlations between variables were less than 0.5. Because bivariate tests showed no statistically significant differences between all media use variables across mental health, all media use variables were entered into subsequent models as control variables. Model fit chi-square statistics indicated good fit (*P*<.05). Adjusted predicted means from linear regression models were also analyzed across subsamples of symptoms (no-mild vs moderate-severe). Figures plotting the adjusted predicted means for all outcomes are presented in the main results (see Tables S1-S4 in Multimedia Appendix 1 for complete linear regression model results). All results were considered significant at *P*<.05 and obtained using StataSEVs.18.

### Ethical Considerations

The study was approved by the institutional review board at Texas A&M University. All study participants provided informed consent and assent and received a US $5 gift card for their time and effort to complete the study. All study data are anonymous and deidentified.

## Results

### Media-Based Health Information Scanning

Table 2 displays the adjusted predicted means of all outcomes, including media-based mental and physical health information scanning across diverse language, cultural, and technological media types, including legacy versus internet-based and social media. In the full sample of Latinx adolescents and subsamples of mental health symptoms, mental and physical health information scanning occurred most often in general English-language media, followed by Latinx-tailored and Spanish-language media. Similar patterns were found for social media.

**Figure 1:**
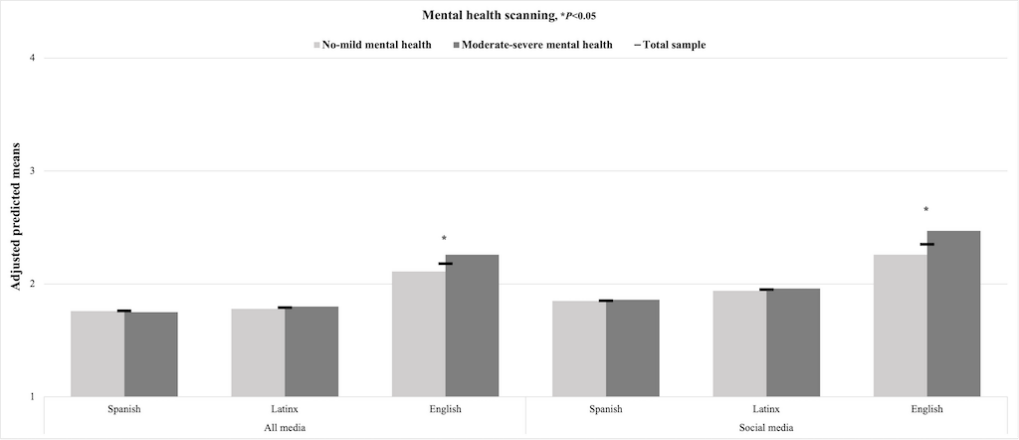
Adjusted predicted means of mental health content scanning in all media in the Latinx youth full sample and subsamples by mental health symptoms (N=701), 2021.

**Figure 2:**
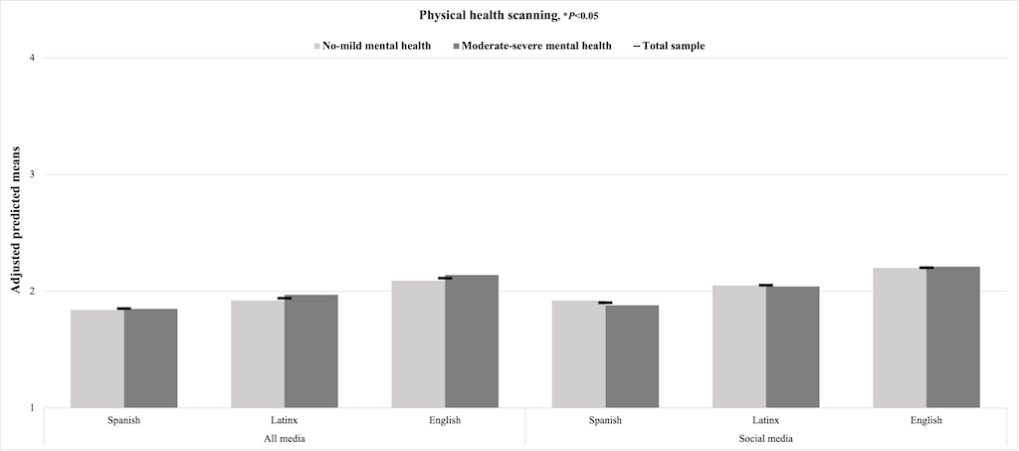
Adjusted predicted means of physical health content scanning in all media in the Latinx youth full sample and subsamples by mental health symptoms (N=701), 2021.

Figure 1 displays the adjusted predicted means of mental and physical health information scanning in the full sample of Latinx adolescents and subsamples of mental health symptoms. No statistically significant differences in mental and physical health information scanning were observed for Latinx-tailored or Spanish media and social media outcomes across subsamples of symptoms. However, Latinx adolescents with moderate-severe symptoms obtained mental health information through scanning English media and social media significantly more often than those with no-mild symptoms (*P*<.05; Figure 1). In contrast, no significant differences in physical health information scanning outcomes were found across mental health (Figure 2).

**Table 2:** Adjusted predicted means from simple linear regression models of mental health and physical health scanning and seeking outcomes in the Latinx youth full sample and subsamples of mental health symptoms (N=701), 2021.

Outcomes					Full sample, margin (95% CI)		No-mild mental health symptoms subsample, margin (95% CI)		Moderate-severe mental health symptoms subsample, margin (95% CI)
**Mental health scanning**									
	**All media**								
		Spanish		1.76 (1.71-1.81)		1.76 (1.7-1.83)		1.75 (1.67-1.82)	
		Latinx		1.79 (1.74-1.84)		1.78 (1.71-1.85)		1.8 (1.72-1.88)	
		English^a^		2.18 (2.12-2.24)		2.11 (2.03-2.19)		2.26 (2.17-2.36)	
	**Social media**								
		Spanish		1.85 (1.79-1.92)		1.85 (1.76-1.94)		1.86 (1.76-1.95)	
		Latinx		1.95 (1.88-2.02)		1.94 (1.85-2.04)		1.96 (1.85-2.06)	
		English^a^		2.35 (2.28-2.43)		2.26 (2.15-2.37)		2.47 (2.35-2.59)	
**Physical health scanning**									
	**All media**								
		Spanish		1.85 (1.79-1.9)		1.84 (1.77-1.91)		1.85 (1.77-1.93)	
		Latinx		1.94 (1.89-2)		1.92 (1.85-2)		1.97 (1.88-2.05)	
		English		2.11 (2.05-2.17)		2.09 (2-2.17)		2.14 (2.05-2.23)	
	**Social media**								
		Spanish		1.9 (1.84-1.96)		1.92 (1.83-2)		1.88 (1.78-1.98)	
		Latinx		2.05 (1.98-2.11)		2.05 (1.95-2.14)		2.04 (1.94-2.15)	
		English		2.2 (2.13-2.28)		2.2 (2.09-2.3)		2.21 (2.09-2.33)	
**Mental health seeking**									
	**All media**								
		Spanish		1.7 (1.65-1.75)		1.68 (1.62-1.75)		1.73 (1.65-1.8)	
		Latinx^a^		1.92 (1.87-1.96)		1.87 (1.81-1.93)		1.98 (1.91-2.05)	
		English^a^		2.01 (1.96-2.06)		1.94 (1.87-2.01)		2.1 (2.02-2.18)	
	**Media type**								
		TV, book, radio		1.68 (1.64-1.72)		1.65 (1.59-1.71)		1.72 (1.65-1.78)	
		Internet, social media^a^		2.17 (2.12-2.22)		2.09 (2.03-2.16)		2.27 (2.19-2.34)	
	**Informal and formal**								
		Family, friend^a^		2.45 (2.37-2.52)		2.29 (2.19-2.39)		2.64 (2.53-2.75)	
		Health care professional^a^		1.77 (1.7-1.83)		1.69 (1.6-1.79)		1.86 (1.76-1.96)	
**Physical health seeking**									
	**All media**								
		Spanish		1.8 (1.75-1.85)		1.77 (1.7-1.84)		1.83 (1.76-1.91)	
		Latinx^a^		1.86 (1.81-1.91)		1.81 (1.74-1.88)		1.93 (1.85-2.01)	
		English		1.92 (1.87-1.97)		1.87 (1.8-1.94)		1.97 (1.89-2.05)	
	**Media type**								
		TV, book, radio		1.69 (1.65-1.74)		1.67 (1.61-1.74)		1.72 (1.65-1.79)	
		Internet, social media^a^		2.1 (2.05-2.16)		2.03 (1.96-2.11)		2.19 (2.11-2.28)	
**Informal and formal**									
		Family, friend^a^		2.46 (2.39-2.53)		2.36 (2.26-2.46)		2.59 (2.48-2.7)	
		Health care professional		1.86 (1.8-1.93)		1.82 (1.72-1.91)		1.92 (1.81-2.03)	

^a^*P*<.05 for significant differences across subsamples of mental health symptoms.

^b^All models adjusted for age, sex, nativity, ethnic origin, race, household income, ethnic attachment, acculturation, and Spanish, Latinx, and English media use; models inclusive of the full sample also adjusted for mental health symptoms while models focused on the subsamples of mental health symptoms did not.

### Media-Based Health Information Seeking

Table 2 also displays the adjusted predicted means of the media-based mental and physical health information-seeking outcomes across diverse language, cultural, and technological media types, including legacy versus internet-based and social media. In the full sample of Latinx adolescents and subsamples of mental health symptoms, mental and physical health information seeking in all media types occurred most often in general English-language media, followed by Latinx-tailored English-language media and Spanish-language media.

In terms of media type, the full sample of Latinx adolescents and both mental health subsamples reported seeking mental and physical health information most often in internet-based and social media versus legacy media.

**Figure 3:**
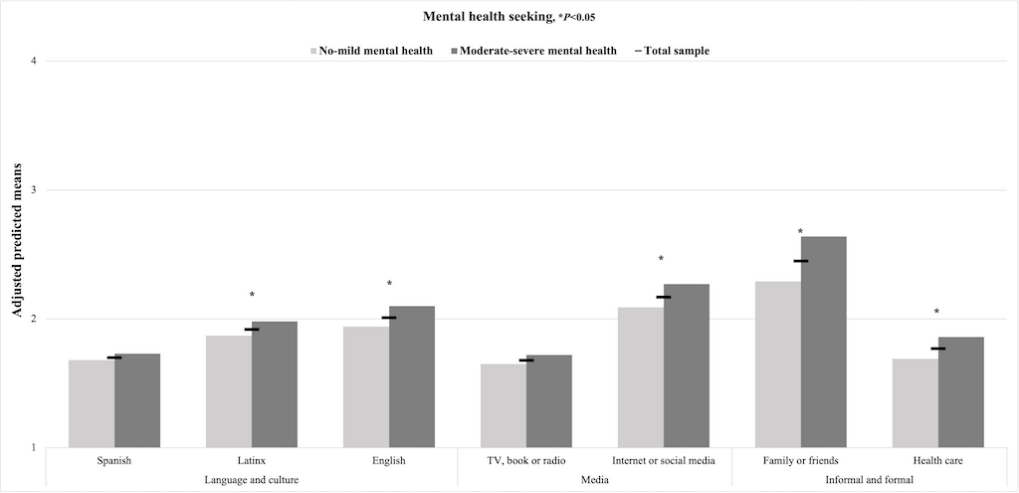
Adjusted predicted means of mental health help-seeking in all media, formal, and informal sectors in the Latinx youth full sample and subsamples by mental health symptoms (N=701), 2021.

**Figure 4:**
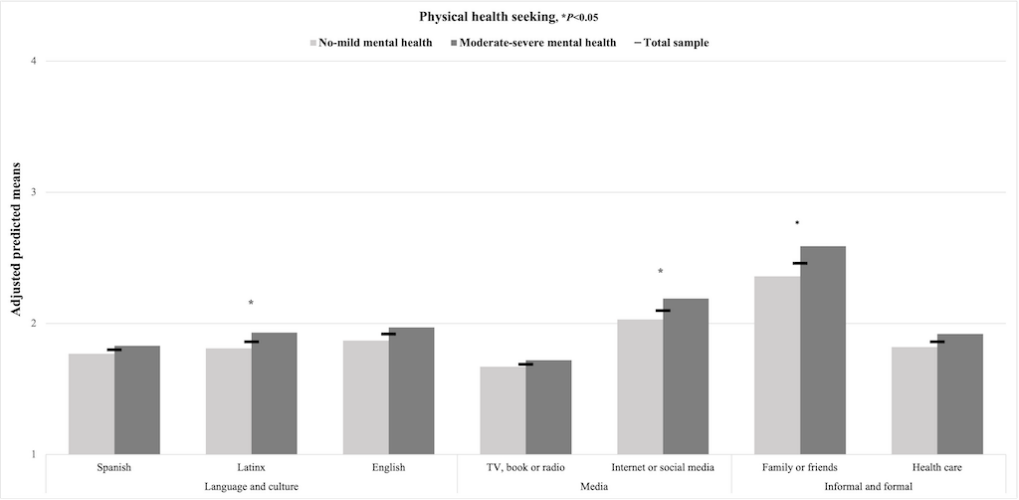
Adjusted predicted means of physical health help-seeking in all media, formal, and informal sectors in the Latinx youth full sample and subsamples by mental health symptoms (N=701), 2021.

Figure 2 displays the adjusted predicted means of mental and physical health information seeking in the full sample of Latinx adolescents and mental health subsamples. No significant differences in mental and physical health information seeking were found for Spanish-language media or legacy media across the mental health subsamples. However, Latinx adolescents with moderate-severe symptoms sought mental health information in Latinx-tailored and nontailored English-language media and internet-based and social media more frequently than those with no-mild symptoms (*P*<.05; Figure 3). Similarly, Latinx adolescents with moderate-severe versus no-mild symptoms sought physical health information in Latinx-tailored English-language media and internet-based and social media more frequently (*P*<.05; Figure 4).

### Formal and Informal Help-Seeking

Table 2 displays the adjusted predicted means of formal (ie, health care providers) and informal (ie, family or friends) help-seeking for mental and physical health problems in the full sample of Latinx adolescents and mental health subsamples. Both the full sample and mental health subsamples sought help for mental and physical health problems more often from family or friends than health care providers.

Figure 2 displays the adjusted predicted means for formal and informal help-seeking for a mental and physical health problem in the full sample of Latinx adolescents and mental health subsamples. Adolescents with moderate-severe versus no-mild symptoms were more likely to seek help from family or friends for both physical or mental health problems (*P*<.05; Figures 3 and 4) and from health care providers for mental health problems only (*P*<.05; Figure 3). Overall, Latinx youth sought help for health problems from family or friends and internet-based and social media more often than from health care providers (Figures 3 and 4).

## Discussion

While data trends depict a national adolescent mental health crisis with simultaneous increases in social media use, our study aimed to provide a more nuanced understanding of these trends across diverse language, cultural, and technological media among Latinx youth. Our study distinguished between mental and physical health, examined diverse language and cultural media domains, distinguished passive and active media exposure (eg, scan vs seek), and accomplished these assessments for both legacy and new digital media.

In terms of passive, casual browsing, overall, our study found that Latinx adolescents with mental health issues were more likely to engage in mental health scanning in English-language legacy and social media, but not in Spanish-language or Latinx-tailored legacy and social media, or for physical health. These patterns may arise because of more prevalent mental health content available in English-language than in Spanish-language media and social media, as previous studies confirm [48]. This is an important finding because as Latinx adolescents with mental health concerns engage with media and social media, they may become more exposed to mental health content, underscoring the need for public mental health action that leverages media to provide quality resources about symptom management and help-seeking. In contrast, passive exposure to mental or physical health content in Spanish-language and Latinx-tailored media did not vary by mental health symptoms possibly because health information is less often available in Spanish and Latinx-media, and not because of a lack of preference or interest in these media among Latinx media consumers [49]. For Latinx families with Spanish and Latinx media preferences, disparate diffusion of health communications actually may help produce health inequities [48].

With respect to the active, purposeful search for health information, our study found significant use of Latinx-tailored and English-language media for mental health information seeking and Latinx-tailored media for physical health information seeking among Latinx adolescents with moderate-severe symptoms. Latinx adolescents with mental health concerns may seek mental health information that is tailored to them and presented in their preferred language and a culturally aligned context. Public health interventionists may take note of this preference, and the availability of tailored, targeted mental and physical health information in media for Latinx adolescent audiences. Finally, family, friends, internet, and social media were more often used for both mental and physical health information than health care professionals, especially among Latinx adolescents with moderate-severe symptoms, indicating a greater preference for more easily accessible informal supports for health problems within the Latinx community. Future studies could examine the role of mistrust of health care experts and providers as sources of health information among Latinx communities to better understand these patterns.

Our study points to Spanish-language and Latinx-tailored media as important sources of health information, with English-language media as the most common source owing to more health information being available there. For example, recent studies of primetime advertising on YouTube TV revealed that English-language TV was more likely to include health promotion advertisements while Spanish-language TV was more likely to include health-adverse advertisements [50]. Inequities in health-related advertising on social media are likely similar though this remains a knowledge gap in the literature. Importantly, our study found that Latinx media is an important source of mental health information for those experiencing mental illness; thus, Latinx media may serve as an important resource for learning about and seeking support for mental health conditions. To this point, media content with greater immigrant community representation and storytelling with respect to mental and physical health issues would be important to foster in future media programming and content curation [38,51].

Findings from this growing body of literature must be implemented into public health policy and practice as Spanish is a global power language and the most common non-English language spoken in the United States. The United States also has the second-largest population of Spanish speakers worldwide behind Mexico. Finally, Latinx populations tend to use social media more than all other major social groups in the United States despite structural inequities in content moderation of Latinx media [23].

This study has some limitations. First, as Latinx populations are dynamic, especially with respect to media and linguistic preferences and behaviors, longitudinal versus cross-sectional assessments could improve causal inference among variables and help assess changes to media content over time such as media campaigns for health promotion. Second, related dimensions of trust and comprehension of health information and perceived health concerns would be important to ascertain. For example, since mental health content is inequitable across language or cultural media, it may influence perceived mental health among English-language media consumers depending on trust and comprehension levels in the content [46]. Third, the study relied on self-reported participant recall of their behaviors. However, recall bias is likely minimal and nondifferential across the subgroups by mental health as the measures asked highly relevant information about oneself that affects daily life. Relatedly, our survey-based research is unable to assess the role of social media algorithms, which influence user consumption. Finally, the study sample was recruited from a proprietary, survey panel; thus, details regarding responses among its volunteers are undisclosed which could potentially lead to bias. To address this concern, we found that the achieved study sample is robust to national data on Latinx populations while also oversampling understudied Latinx groups, as previously reported [46].

In summary, our study demonstrates a more diverse portrait of adolescent media use in the United States, specifically Latinx adolescents who have diasporic, global ties to diverse language, cultural, and technological media. While policies and public attitudes about media and social media tend to generalize these issues, our study suggests potential contexts in which diverse language, cultural, and technological media can be an important, beneficial source of health information for Latinx adolescents. Current scrutiny about the effects of social media use on adolescent health has not sufficiently approached this issue through a health equity lens, which is essential to help inform equitable policies, programs, and practices related to its safe use among adolescents. Diverse adolescent populations will have unique contexts, needs, and assets with respect to the use of diverse language, cultural, and technological media. For Latinx adolescents experiencing mental illness, social media seems to be a useful resource of support, which has implications for other minoritized populations who might find social media as a tool to prevent health risks. Thus, greater access and equity with respect to digital media remain critical to health.

## Data Availability

The dataset generated and analyzed during this study are available from the corresponding author on reasonable request.

## Appendix

Multimedia Appendix 1 Tables with coefficients and 95% confidence intervals B [95% CI] from linear regression models assessing media outcomes in the Latinx youth full sample; N=701, 2021.

## Abbreviations

PHQ-4: Patient Health Questionnaire-4
