# Supplementary material for: Health Information Scanning and Seeking in Diverse Language, Cultural and Technological Media Among Latinx Adolescents: Cross-Sectional Study
Source: J Med Internet Res. 2025 Mar 5;27:e64672. doi: 10.2196/64672 (PMC11923458; doi:10.2196/64672)
Supplement: Multimedia Appendix 1 [file jmir_v27i1e64672_app1.pdf]

Appendix

Table S1. Coefficient and 95% confidence intervals B [95% CI] from linear regression models assessing mental health information scanning in the Latinx youth full sample; N=701, 2021

Table S2. Coefficient and 95% confidence intervals B [95% CI] from linear regression models assessing physical health information scanning in the Latinx youth full sample; N=701, 2021

Table S3. Coefficient and 95% confidence intervals B [95% CI] from linear regression models assessing mental health information seeking in the Latinx youth full sample; N=701, 2021

Table S4. Coefficient and 95% confidence intervals B [95% CI] from linear regression models assessing physical health information seeking in the Latinx youth full sample; N=701, 2021

Table S1. Coefficient and 95% confidence intervals B [95% CI] from linear regression models assessing mental health information scanning in the Latinx youth full sample; N=701, 2021

Predictor Variables	All Media			Social Media		
	Spanish	Latinx	English	Spanish	Latinx	English
	F = 20.53 (21, 648)***	F = 17.21 (21, 648)***	F = 10.13 (21, 648)***	F = 15.77 (21, 648)***	F = 12.22 (21, 648)***	F = 5.01 (21, 648)***
**Age**	0.00 [-0.03, 0.03]	0.01 [-0.02, 0.05]	-0.03 [-0.07, 0.01]	-0.00 [-0.04, 0.04]	0.01 [-0.03, 0.06]	-0.02 [-0.07, 0.04]
**Gender**						
Female	Ref.	Ref.	Ref.	Ref.	Ref.	Ref.
Male	0.07 [-0.06, 0.20]	0.08 [-0.05, 0.22]	0.09 [-0.06, 0.24]	0.06 [-0.10, 0.22]	-0.08 [-0.25, 0.09]	-0.03 [-0.23, 0.17]
**Nativity**						
U.S. born	Ref.	Ref.	Ref.	Ref.	Ref.	Ref.
Foreign born	0.15 [-0.03, 0.32]	0.20 [0.02, 0.38]*	0.08 [-0.12, 0.29]	0.29 [0.07, 0.51]*	0.40 [0.16, 0.63]**	0.10 [-0.17, 0.37]
**Ethnicity**						
Central American	Ref.	Ref.	Ref.	Ref.	Ref.	Ref.
Cuban/Dominican/Other	-0.10 [-0.31, 0.11]	-0.13 [-0.35, 0.08]	0.01 [-0.25, 0.26]	-0.06 [-0.18, 0.77]	-0.17 [-0.46, 0.12]	0.13 [-0.21, 0.46]
Mexican	-0.03 [-0.20, 0.13]	-0.07 [-0.24, 0.09]	0.07 [-0.12, 0.26]	-0.03 [-0.24, 0.17]	-0.05 [-0.27, 0.17]	0.14 [-0.12, 0.39]
Puerto Rican	0.00 [-0.22, 0.23]	0.01 [-0.22, 0.24]	0.14 [-0.13, 0.41]	-0.04 [-0.33, 0.25]	-0.01 [-0.32, 0.30]	0.24 [-0.11, 0.60]
South American	0.04 [-0.20, 0.27]	-0.10 [-0.34, 0.14]	0.22 [-0.06, 0.49]	-0.07 [-0.36, 0.22]	-0.13 [-0.45, 0.19]	0.11 [-0.25, 0.48]
**Race**						
Black/African American	Ref.	Ref.	Ref.	Ref.	Ref.	Ref.
American Indian/Alaskan Native	0.18 [-0.20, 0.55]	0.45 [0.06, 0.83]*	0.49 [0.04, 0.94]*	0.30 [-0.18, 0.77]	0.34 [-0.17, 0.85]	0.55 [-0.04, 1.14]
Multiracial	-0.13 [-0.40, 0.14]	0.17 [-0.10, 0.45]	0.22 [-0.10, 0.54]	0.03 [-0.31, 0.37]	0.27 [-0.09, 0.64]	0.36 [-0.06, 0.78]
Other	-0.20 [-0.44, 0.04]	0.07 [-0.17, 0.32]	0.14 [-0.15, 0.43]	-0.14 [-0.45, 0.16]	0.15 [-0.18, 0.48]	0.16 [-0.22, 0.54]
White	-0.13 [-0.37, 0.12]	0.12 [-0.12, 0.37]	0.20 [-0.08, 0.49]	0.00 [-0.30, 0.31]	0.25 [-0.08, 0.58]	0.31 [-0.07, 0.69]
**Household income**						
$75,000+	Ref.	Ref.	Ref.	Ref.	Ref.	Ref.
$35,000 — $74,999	-0.05 [-0.22, 0.13]	0.00 [-0.17, 0.18]	0.06 [-0.15, 0.26]	0.09 [-0.13, 0.31]	0.09 [-0.14, 0.33]	0.09 [-0.18, 0.36]
$20,000 — $34,999	0.13 [-0.06, 0.31]	0.13 [-0.06, 0.32]	0.05 [-0.17, 0.27]	0.12 [-0.11, 0.35]	0.02 [-0.23, 0.27]	0.04 [-0.25, 0.33]
$0 — $19,999	0.11 [-0.06, 0.27]	0.18 [0.01, 0.35]*	0.06 [-0.13, 0.26]	0.13 [-0.08, 0.34]	0.16 [-0.07, 0.38]	-0.02 [-0.28, 0.24]
**Latino ethnic attachment**	-0.01 [-0.01, 0.00]	-0.004 [-0.01, 0.00]	0.00 [-0.01, 0.01]	-0.01 [-0.02, 0.00]	0.00 [-0.01, 0.01]	0.01 [-0.01, 0.02]
**Spanish acculturation**	-0.01 [-0.02, 0.00]	-0.02 [-0.03, -0.01]**	-0.01 [-0.02, 0.00]	0.00 [-0.01, 0.02]	-0.02 [-0.03, -0.00]*	-0.01 [-0.02, 0.01]
**English acculturation**	-0.00 [-0.02, 0.02]	0.03 [0.01, 0.05]**	0.03 [0.00, 0.05]*	0.00 [-0.02, 0.03]	0.05 [0.02, 0.07]**	0.03 [-0.01, 0.06]
**Spanish media use**	0.46 [0.38, 0.54]***	0.26 [0.18, 0.34]***	0.17 [0.07, 0.26]***	0.46 [0.36, 0.55]***	0.21 [0.12, 0.32]***	0.09 [-0.03, 0.21]
**Latinx media use**	0.09 [0.02, 0.16]*	0.31 [0.24, 0.39]***	0.21 [0.13, 0.30]***	0.10 [0.01, 0.19]*	0.39 [0.30, 0.49]***	0.21 [0.10, 0.32]***
**English media use**	0.01 [-0.06, 0.07]	-0.007 [-0.07, 0.06]	0.18 [0.11, 0.25]***	0.03 [-0.05, 0.11]	-0.02 [-0.11, 0.06]	0.16 [0.06, 0.25]**
**Mental health symptoms**						
None/Mild	Ref.	Ref.	Ref.	Ref.	Ref.	Ref.
Moderate/Severe	-0.02 [-0.12, 0.09]	0.02 [-0.09, 0.13]	0.15 [0.03, 0.28]*	0.01 [-0.13, 0.14]	0.01 [-0.13, 0.15]	0.22 [0.05, 0.38]*
**_cons**	0.83 [-0.11, 1.78]	-0.58 [-1.55, 0.38]	-0.02 [-1.14, 1.10]	0.35 [-0.85, 1.54]	-1.44 [-2.72, -0.15]*	-0.08 [-1.55, 1.40]

NOTES: All models adjust for age, sex, nativity, ethnic origin, race, household income, ethnic attachment, acculturation, media use, and mental health symptoms; *p<0.05, **p<0.01, ***p<0.001

Table S2. Coefficient and 95% confidence intervals B [95% CI] from linear regression models assessing physical health information scanning in the Latinx youth full sample; N=701, 2021

Predictor Variables	All Media			Social Media		
	Spanish	Latinx	English	Spanish	Latinx	English
	F = 23.44 (21, 648)***	F = 19.30 (21, 648)***	F = 11.78 (21, 648)***	F =16.84 (21, 648)***	F = 12.83 (21, 648)***	F = 6.50 (21, 648)***
**Age**	-0.01 [-0.05, 0.02]	-0.01 [-0.05, 0.02]	-0.03 [-0.07, 0.01]	-0.01 [-0.05, 0.04]	-0.01 [-0.05, 0.04]	-0.02 [-0.07, 0.04]
**Gender**						
Female	Ref.	Ref.	Ref.	Ref.	Ref.	Ref.
Male	0.11 [-0.02, 0.24]	0.21 [0.07, 0.34]**	0.19 [0.04, 0.34]*	0.11 [-0.05, 0.27]	0.18 [0.00, 0.35]*	0.13 [-0.06, 0.33]
**Nativity**						
U.S. born	Ref.	Ref.	Ref.	Ref.	Ref.	Ref.
Foreign born	0.12 [-0.05, 0.30]	0.11 [-0.08, 0.30]	0.06 [-0.15, 0.26]	0.17 [-0.05, 0.39]	0.22 [-0.02, 0.46]	0.19 [-0.08, 0.46]
**Ethnicity**						
Central American	Ref.	Ref.	Ref.	Ref.	Ref.	Ref.
Cuban/Dominican/Other	-0.16 [-0.38, 0.06]	-0.08 [-0.31, 0.15]	0.12 [-0.13, 0.37]	-0.17 [-0.44, 0.10]	-0.17 [-0.46, 0.12]	0.07 [-0.25, 0.40]
Mexican	-0.03 [-0.19, 0.14]	-0.01 [-0.19, 0.16]	0.09 [-0.10, 0.28]	0.01 [-0.19, 0.22]	0.07 [-0.15, 0.29]	0.19 [-0.05, 0.44]
Puerto Rican	0.01 [-0.22, 0.25]	0.11 [-0.14, 0.35]	0.17 [-0.10, 0.43]	0.17 [-0.12, 0.45]	0.25 [-0.06, 0.56]	0.19 [-0.15, 0.54]
South American	0.06 [-0.18, 0.30]	0.03 [-0.22, 0.29]	0.21 [-0.07, 0.49]	0.11 [-0.18, 0.41]	-0.05 [-0.37, 0.27]	0.23 [-0.13, 0.58]
**Race**						
Black/African American	Ref.	Ref.	Ref.	Ref.	Ref.	Ref.
American Indian/Alaskan Native	0.16 [-0.22, 0.55]	0.45 [0.04, 0.86]*	0.52 [0.07, 0.97]*	0.11 [-0.37, 0.58]	0.29 [-0.22, 0.81]	0.24 [-0.34, 0.81]
Multiracial	-0.13 [-0.41, 0.15]	-0.08 [-0.37, 0.21]	0.10 [-0.21, 0.42]	-0.07 [-0.40, 0.27]	-0.13 [-0.49, 0.24]	0.04 [-0.37, 0.45]
Other	-0.15 [-0.40, 0.10]	-0.02 [-0.28, 0.25]	0.09 [-0.20, 0.38]	-0.04 [-0.34, 0.27]	-0.13 [-0.46, 0.20]	-0.06 [-0.43, 0.32]
White	-0.10 [-0.35, 0.15]	0.04 [-0.22, 0.30]	0.07 [-0.22, 0.35]	-0.01 [-0.32, 0.29]	-0.04 [-0.37, 0.29]	-0.07 [-0.44, 0.30]
**Household income**						
$75,000+	Ref.	Ref.	Ref.	Ref.	Ref.	Ref.
$35,000 — $74,999	0.09 [-0.09, 0.26]	0.00 [-0.18, 0.19]	0.10 [-0.10, 0.31]	0.20 [-0.02, 0.41]	-0.05 [-0.29, 0.18]	0.17 [-0.09, 0.43]
$20,000 — $34,999	0.13 [-0.05, 0.32]	0.22 [0.02, 0.42]*	0.14 [-0.08, 0.35]	0.20 [-0.03, 0.43]	0.11 [-0.14, 0.36]	0.12 [-0.16, 0.40]
$0 — $19,999	0.14 [-0.03, 0.31]	0.25 [0.07, 0.43]**	0.05 [-0.15, 0.24]	0.19 [-0.01, 0.40]	0.17 [-0.05, 0.40]	-0.01 [-0.26, 0.24]
**Latino ethnic attachment**	-0.00 [-0.01, 0.01]	-0.00 [-0.01, 0.01]	-0.00 [-0.01, 0.01]	-0.01 [-0.01, 0.01]	0.00 [-0.01, 0.01]	0.00 [-0.01, 0.02]
**Spanish acculturation**	0.00 [-0.01, 0.01]	-0.01 [-0.03, -0.00]**	-0.01 [-0.02, 0.00]	0.01 [0.0, 0.03]*	-0.01 [-0.03, 0.00]	-0.01 [-0.02, 0.01]
**English acculturation**	-0.01 [-0.03, 0.01]	0.01 [-0.01, 0.03]	0.01 [-0.02, 0.03]	0.00 [-0.02, 0.3]	0.02 [-0.01, 0.04]	0.01 [-0.02, 0.04]
**Spanish media use**	0.42 [0.34, 0.50]***	0.24 [0.16, 0.33]***	0.21 [0.12, 0.30]***	0.39 [0.30, 0.49]***	0.24 [0.13, 0.35]***	0.19 [0.07, 0.31]**
**Latinx media use**	0.13 [0.06, 0.20]***	0.33 [0.25, 0.40]***	0.21 [0.12, 0.29]***	0.12 [0.03, 0.21]*	0.31 [0.21, 0.40]***	0.16 [0.05, 0.26]**
**English media use**	0.04 [-0.03, 0.10]	0.10 [0.03, 0.17]**	0.20 [0.13, 0.27]***	0.03 [-0.05, 0.10]	0.13 [0.05, 0.22]**	0.23 [0.13, 0.32]***
**Mental health symptoms**						
None/Mild	Ref.	Ref.	Ref.	Ref.	Ref.	Ref.
Moderate/Severe	0.00 [-0.10, 0.11]	0.04 [-0.07, 0.16]	0.06 [-0.07, 0.18]	-0.04 [-0.17, 0.09]	-0.00 [-0.15, 0.14]	0.01 [-0.15, 0.17]
**_cons**	0.87 [-0.10, 1.83]	0.16 [-0.86, 1.19]	0.65 [-0.47, 1.77]	-0.14 [-1.32, 1.05]	-0.18 [-1.47, 1.11]	0.24 [-1.20, 1.69]

NOTES: All models adjust for age, sex, nativity, ethnic origin, race, household income, ethnic attachment, acculturation, media use, and mental health symptoms; *p<0.05, **p<0.01, ***p<0.001

Table S3. Coefficient and 95% confidence intervals B [95% CI] from linear regression models assessing mental health information seeking in the Latinx youth full sample; N=701, 2021

Predictor Variables	Language/Culture Media			Media Type		Informal/Formal	
	**Spanish**	**Latinx**	**English**	**TV/Book/Radio**	**Internet/Social Media**	**Family/Friend**	**Healthcare**
	F = 13.72 (21, 648)***	F = 14.08 (21, 648)***	F = 9.47 (21, 648)***	F = 13.41 (21, 648)***	F =12.58 (21, 648)***	F = 5.54 (21, 648)***	F = 3.45 (21, 648)***
**Age**	-0.01 [-0.04, 0.02]	-0.01 [-0.04, 0.02]	-0.03 [-0.06, 0.01]	-0.02 [-0.05, 0.01]	-0.01 [-0.04, 0.02]	-0.02 [-0.07, 0.03]	-0.01 [-0.05, 0.04]
**Gender**							
Female	Ref.	Ref.	Ref.	Ref.	Ref.	Ref.	Ref.
Male	0.11 [-0.02, 0.23]	0.02 [-0.10, 0.13]	0.08 [-0.05, 0.21]	0.09 [-0.02, 0.20]	0.03 [-0.09, 0.16]	-0.06 [-0.24, 0.12]	-0.00 [-0.17, 0.17]
**Nativity**							
U.S. born	Ref.	Ref.	Ref.	Ref.	Ref.	Ref.	Ref.
Foreign born	-0.01 [-0.18, 0.16]	-0.02 [-0.18, 0.13]	-0.02 [-0.20, 0.15]	0.01 [-0.14, 0.16]	-0.06 [-0.23, 0.11]	0.01 [-0.24, 0.25]	0.07 [-0.16, 0.30]
**Ethnicity**							
Central American	Ref.	Ref.	Ref.	Ref.	Ref.	Ref.	Ref.
Cuban/Dominican/Other	-0.09 [-0.30, 0.11]	-0.03 [-0.22, 0.17]	-0.08 [-0.30, 0.14]	-0.00 [-0.18, 0.18]	-0.16 [-0.38, 0.05]	-0.07 [-0.38, 0.23]	0.01 [-0.28, 0.30]
Mexican	-0.01 [-0.17, 0.14]	-0.05 [-0.19, 0.10]	-0.09 [-0.26, 0.07]	0.00 [-0.14, 0.14]	-0.13 [-0.29, 0.03]	-0.23 [-0.46, -0.00]*	-0.07 [-0.29, 0.14]
Puerto Rican	0.03 [-0.18, 0.25]	-0.08 [-0.28, 0.12]	-0.04 [-0.27, 0.19]	0.06 [-0.13, 0.25]	-0.16 [-0.39, 0.06]	0.08 [-0.24, 0.40]	-0.10 [-0.41, 0.20]
South American	-0.11 [-0.33, 0.11]	-0.18 [-0.39, 0.03]	-0.25 [-0.048, -0.01]*	-0.14 [-0.34, 0.06]	-0.24 [-0.47, -0.01]*	-0.03 [-0.36, 0.30]	-0.06 [-0.37, 0.26]
**Race**							
Black/African American	Ref.	Ref.	Ref.	Ref.	Ref.	Ref.	Ref.
American Indian/Alaskan Native	0.08 [-0.27, 0.44]	0.13 [-0.21, 0.47]	0.27 [-0.11, 0.65]	0.11 [-0.21, 0.43]	0.24 [-0.14, 0.61]	0.68 [0.15, 1.21]*	0.40 [-0.11, 0.90]
Multiracial	-0.00 [-0.26, 0.25]	-0.22 [-0.47, 0.02]	-0.03 [-0.30, 0.24]	-0.11 [-0.34, 0.12]	-0.04 [-0.31, 0.23]	0.19 [-0.19, 0.57]	0.06 [-0.30, 0.42]
Other	-0.12 [-0.35, 0.11]	-0.15 [-0.37, 0.07]	-0.05 [-0.30, 0.19]	-0.11 [-0.32, 0.10]	-0.10 [-0.34, 0.14]	0.06 [-0.28, 0.40]	-0.06 [-0.38, 0.27]
White	-0.11 [-0.34, 0.12]	-0.22 [-0.44, -0.01]*	-0.02 [-0.26, 0.23]	-0.10 [-0.30, 0.11]	-0.15 [-0.36, 0.09]	0.06 [-0.28, 0.40]	-0.01 [-0.34, 0.31]
**Household income**							
$75,000+	Ref.	Ref.	Ref.	Ref.	Ref.	Ref.	Ref.
$35,000 — $74,999	-0.07 [-0.23, 0.10]	0.01 [-0.14, 0.17]	-0.13 [-0.30, 0.05]	-0.09 [-0.24, 0,05]	-0.01 [-0.18, 0.16]	0.00 [-0.24, 0.24]	0.07 [-0.16, 0.30]
$20,000 — $34,999	0.06 [-0.12, 0.23]	0.10 [-0.06, 0.27]	0.02 [-0.16, 0.21]	0.04 [-0.11, 0.20]	0.09 [-0.10, 0.27]	-0.03 [-0.29, 0.23]	0.09 [-0.16, 0.33]
$0 — $19,999	0.07 [-0.09, 0.22]	0.09 [-0.06, 0.24]	-0.06 [-0.23, 0.11]	0.05 [-0.09, 0.19]	0.01 [-0.15, 0.17]	0.07 [-0.17, 0.30]	0.06 [-0.16, 0.28]
**Latino ethnic attachment**	-0.00 [-0.01, 0.00]	0.01 [0.00, 0.01]*	-0.00 [-0.01, 0.01]	-0.00 [-0.01, 0.00]	0.01 [0.00, 0.02]*	0.01 [0.00, 0.02]	0.00 [-0.01, 0.01]
**Spanish acculturation**	-0.00 [-0.01, 0.01]	-0.01 [-0.02, -0.01]**	-0.01 [-0.02, 0.00]	-0.01 [-0.02, -0.00]*	-0.00 [-0.01, 0.01]	-0.02 [-0.04, -0.01]**	-0.02 [-0.03, -0.01]**
**English acculturation**	0.01 [-0.01, 0.02]	0.02 [0.00, 0.04]*	0.02 [0.00, 0.04]*	0.01 [-0.00, 0.03]	0.02 [-0.00, 0.04]	0.02 [-0.00, 0.05]	0.02 [-0.01, 0.04]
**Spanish media use**	0.32 [0.25, 0.40]***	0.24 [0.17, 0.32]***	0.17 [0.09, 0.25]***	0.26 [0.19, 0.32]***	0.23 [0.15, 0.30]***	0.13 [0.02, 0.24]*	0.22 [0.12, 0.33]***
**Latinx media use**	0.10 [0.03, 0.16]**	0.16 [0.10, 0.22]***	0.15 [0.08, 0.22]***	0.13 [0.07, 0.19]***	0.15 [0.08, 0.22]***	0.10 [0.00, 0.20]*	0.05 [-0.05, 0.14]
**English media use**	0.03 [-0.02, 0.09]	0.08 [0.02, 0.13]**	0.14 [0.07, 0.20]***	0.08 [0.02, 0.13]**	0.09 [0.03, 0.15]**	0.13 [0.04, 0.22]**	0.10 [0.02, 0.18]*
**Mental health symptoms**							
None/Mild	Ref.	Ref.	Ref.	Ref.	Ref.	Ref.	Ref.
Moderate/Severe	0.05 [-0.05, 0.15]	0.12 [0.02, 0.21]*	0.16 [0.05, 0.27]	0.07 [-0.02, 0.16]	0.17 [0.07, 0.27]**	0.35 [0.21, 0.50]***	0.17 [0.03, 0.31] *
**_cons**	0.60 [-0.30, 1.49]	0.38 [-0.48, 1.23]	0.72 [-0.24, 1.67]	0.69 [-0.12, 1.49]	0.38 [-0.56, 1.31]	0.99 [-0.34, 2.32]	-0.71 [-0.56, 1.97]

NOTES: All models adjust for age, sex, nativity, ethnic origin, race, household income, ethnic attachment, acculturation, media use, and mental health symptoms; *p<0.05, **p<0.01, ***p<0.001

Table S4. Coefficient and 95% confidence intervals B [95% CI] from linear regression models assessing physical health information seeking in the Latinx youth full sample; N=701, 2021

Predictor Variables	Language/Culture Media			Media Type		Informal/Formal	
	**Spanish**	**Latinx**	**English**	**TV/Book/Radio**	**Internet/Social Media**	**Family/Friend**	**Healthcare**
	F = 12.27 (21, 648)***	F = 11.01 (21, 648)***	F = 8.38 (21, 648)***	F = 10.77 (21, 648)***	F = 10.59 (21, 648)***	F = 4.99 (21, 648)***	F = 4.83 (21, 648)***
**Age**	-0.01 [0.04, 0.03]	-0.01 [-0.05, 0.02]	0.00 [-0.03, 0.04]	-0.01 [-0.04, 0.02]	0.00 [-0.03, 0.04]	-0.00 [-0.05, 0.05]	0.02 [-0.03, 0.06]
**Gender**							
Female	Ref.	Ref.	Ref.	Ref.	Ref.	Ref.	Ref.
Male	0.07 [-0.05, 0.19]	0.09 [-0.04, 0.22]	0.11 [-0.02, 0.23]	0.11 [-0.01, 0.23]	0.06 [-0.07, 0.20]	0.05 [-0.13, 0.24]	-0.03 [-0.21, 0.15]
**Nativity**							
U.S. born	Ref.	Ref.	Ref.	Ref.	Ref.	Ref.	Ref.
Foreign born	0.05 [-0.12, 0.22]	0.06 [-0.12, 0.24]	0.05 [-0.13, 0.22]	0.06 [-0.10, 0.22]	0.04 [-0.14, 0.23]	0.12 [-0.13, 0.38]	0.04 [-0.21, 0.28]
**Ethnicity**							
Central American	Ref.	Ref.	Ref.	Ref.	Ref.	Ref.	Ref.
Cuban/Dominican/Other	0.02 [-0.19, 0.22]	-0.05 [-0.26, 0.17]	-0.05 [-0.27, 0.16]	0.05 [-0.15, 0.25]	-0.15 [-0.38, 0.08]	-0.02 [-0.34, 0.29]	0.35 [0.06, 0.65]*
Mexican	-0.01 [-0.17, 0.15]	-0.04 [-0.20, 0.13]	-0.05 [-0.21, 0.12]	0.01 [-0.14, 0.16]	-0.09 [-0.26, 0.08]	-0.11 [-0.35, 0.12]	0.12 [-0.11, 0.34]
Puerto Rican	0.06 [-0.15, 0.28]	0.07 [-0.16, 0.30]	0.09 [-0.14, 0.31]	0.16 [-0.05, 0.38]	-0.06 [-0.30, 0.18]	0.05 [-0.28, 0.38]	-0.17 [-0.48, 0.15]
South American	-0.22 [-0.44, 0.01]	-0.18 [-0.41, 0.06]	-0.18 [-0.41, 0.06]	-0.16 [-0.38, 0.06]	-0.24 [-0.48, 0.01]	-0.18 [-0.52, 0.16]	0.14 [-0.18, 0.47]
**Race**							
Black/African American	Ref.	Ref.	Ref.	Ref.	Ref.	Ref.	Ref.
American Indian/Alaskan Native	0.12 [-0.24, 0.49]	-0.06 [-0.44, 0.32]	0.03 [-0.35, 0.41]	-0.03 [-0.38, 0.32]	0.12 [-0.28, 0.51]	0.28 [-0.27, 0.83]	0.33 [-0.20, 0.85]
Multiracial	0.03 [-0.23, 0.29]	-0.23 [-0.50, 0.04]	-0.03 [-0.30, 0.24]	-0.08 [-0.33, 0.17]	-0.08 [-0.36, 0.21]	0.02 [-0.37, 0.42]	0.31 [-0.06, 0.68]
Other	-0.03 [-0.27, 0.20]	-0.24 [-0.48, 0.01]	-0.04 [-0.29, 0.20]	-0.11 [-0.33, 0.12]	-0.10 [-0.36, 0.15]	-0.06 [-0.42, 0.30]	-0.04 [-0.38, 0.30]
White	-0.05 [-0.28, 0.18]	-0.22 [-0.46, 0.03]	-0.04 [-0.28, 0.20]	-0.08 [-0.31, 0.14]	-0.13 [-0.39, 0.12]	-0.05 [-0.41, 0.31]	-0.03 [-0.37, 0.31]
**Household income**							
$75,000+	Ref.	Ref.	Ref.	Ref.	Ref.	Ref.	Ref.
$35,000 — $74,999	-0.01 [-0.17, 0.16]	0.04 [-0.13, 0.21]	-0.01 [-0.19, 0.16]	-0.04 [-0.20, 0.12]	0.08 [-0.10, 0.26]	-0.17 [-0.42, 0.08]	0.06 [-0.18, 0.30]
$20,000 — $34,999	0.03 [-0.15, 0.21]	0.11 [-0.07, 0.30]	0.08 [-0.10, 0.27]	0.05 [-0.12, 0.22]	0.12 [-0.08, 0.31]	-0.18 [0.45, 0.09]	-0.05 [-0.31, 0.20]
$0 — $19,999	0.08 [-0.08, 0.24]	0.15 [-0.01, 0.32]	0.02 [-0.14, 0.19]	0.07 [-0.08, 0.23]	0.10 [-0.07, 0.28]	0.00 [-0.24, 0.24]	0.03 [-0.20, 0.26]
**Latino ethnic attachment**	-0.00 [-0.01, 0.01]	0.00 [-0.00, 0.01]	0.00 [-0.00, 0.01]	-0.00 [-0.01, 0.00]	0.01 [0.00, 0.02]*	0.01 [0.00, 0.03]*	0.01 [-0.00, 0.02]
**Spanish acculturation**	-0.01 [-0.02, 0.00]	-0.01 [-0.02, 0.00]	-0.01 [-0.02, -0.00]*	-0.01 [-0.02, -0.00]**	-0.00 [-0.01, 0.01]	-0.01 [-0.03, 0.00]	-0.02 [-0.03, -0.00]*
**English acculturation**	0.00 [-0.02, 0.02]	0.02 [-0.00, 0.04]	0.01 [-0.01, 0.03]	0.01 [-0.01, 0.03]	0.01 [-0.01, 0.03]	0.04 [0.02, 0.07]**	0.03 [-0.00, 0.05]
**Spanish media use**	0.29 [0.21, 0.36]***	0.21 [0.13, 0.29]***	0.21 [0.13, 0.28]***	0.24 [0.16, 0.31]***	0.23 [0.15, 0.32]***	0.21 [0.09, 0.32]***	0.17 [0.06, 0.27]**
**Latinx media use**	0.13 [0.06, 0.20]***	0.18 [0.11, 0.26]***	0.09 [0.02, 0.16]*	0.13 [0.06, 0.20]***	0.14 [0.06, 0.21]***	0.10 [-0.00, 0.21]	0.13 [0.03, 0.22]*
**English media use**	0.03 [-0.03, 0.09]	0.07 [0.00, 0.13]*	0.16 [0.09, 0.22]***	0.09 [0.03, 0.15]**	0.08 [0.02, 0.15]*	0.09 [-0.00, 0.18]	0.10 [0.01, 0.18]*
**Mental health symptoms**							
None/Mild	Ref.	Ref.	Ref.	Ref.	Ref.	Ref.	Ref.
Moderate/Severe	0.06 [-0.04, 0.16]	0.12 [0.01, 0.23]*	0.10 [-0.01, 0.20]	0.05 [-0.05, 0.15]	0.16 [0.05, 0.27]**	0.23 [0.08, 0.39]**	0.10 [-0.04, 0.25]
**_cons**	0.80 [-0.11, 1.71]	0.42 [-0.54, 1.38]	0.38 [-0.57, 1.32]	0.83 [-0.05, 1.70]	0.09 [-0.91, 1.09]	-0.15 [-1.54, 1.23]	-0.35, [-1.66, 0.96]

NOTES: All models adjust for age, sex, nativity, ethnic origin, race, household income, ethnic attachment, acculturation, media use, and mental health symptoms; *p<0.05, **p<0.01, ***p<0.001
